# Electro-activation of potassium acetate, potassium citrate and calcium lactate: impact on solution acidity, Redox potential, vibrational properties of Raman spectra and antibacterial activity on *E. coli* O157:H7 at ambient temperature

**DOI:** 10.1186/s40064-016-3453-1

**Published:** 2016-10-10

**Authors:** Viacheslav Liato, Steve Labrie, Mohammed Aïder

**Affiliations:** 1 Institute of Nutrition and Functional Foods (INAF), Université Laval, Quebec, QC G1V 0A6 Canada; 2 Department of Soil Sciences and Agri-Food Engineering, Université Laval, Quebec, QC G1V 0A6 Canada; 3 Department of Food Sciences, Université Laval, Quebec, QC G1V 0A6 Canada; 4 Laval University, 2425 Rue de l’Agriculture, Pavillon P. Comtois, Quebec, QC G1V 0A6 Canada

**Keywords:** Electro-activation, Acetate, Citrate, Lactate, Acidity, ORP, Raman, *E. coli* O157:H7

## Abstract

**Aims:**

To study the electro-activation of potassium acetate, potassium citrate and calcium lactate aqueous solutions and to evaluate their antimicrobial effect against *E. coli* O157:H7 at ambient temperature.

**Methods and results:**

Potassium acetate, potassium citrate and calcium lactate aqueous solutions were electrically excited in the anodic compartment of a four sectional electro-activation reactor. Different properties of the electro-activated solutions were measured such as: solutions acidity (pH and titratable), Redox potential and vibrational properties by Raman spectroscopy. Moreover, the antimicrobial activity of these solutions was evaluated against *E. coli* O157:H7. The results showed a pH decrease from 7.07 ± 0.08, 7.53 ± 0.12 and 6.18 ± 0.1 down to 2.82 ± 0.1, 2.13 ± 0.09 and 2.26 ± 0.15, after 180 min of electro-activation of potassium acetate, potassium citrate and calcium lactate solution, respectively. These solutions were characterized by high oxidative ORP of +1076 ± 12, +958 ± 11 and +820 ± 14 mV, respectively. Raman scattering analysis of anolytes showed stretching vibrations of the hydrogen bonds with the major changes within the region of 3410–3430 cm^−1^. These solutions were used against *E. coli* O157:H7 and the results from antimicrobial assays showed high antibacterial effect with a population reduction of ≥6 log CFU/ml within 5 min of treatment.

**Conclusions:**

This study demonstrated the effectiveness of the electro-activation to confer to aqueous solutions of organic salts of highly reactive properties that differ them from their conjugated commercial acids. The electro-activated solutions demonstrated significant antimicrobial activity against *E. coli* O157:H7.

**Significance and impact of study:**

This study opens new possibilities to use electro-activated solutions of salts of weak organic acids as food preservatives to develop safe, nutritive and low heat processed foods.

## Background

Application of organic acids as preservative agents and disinfectants is a common practice in drug, cosmetic and food industries (Rico et al. 2007). Moreover, particular interest for different organic acids is continuously increasing worldwide creating thereby a necessity for production of huge quantities of these chemicals. From economical point of view, it is more important to produce concentrated as opposed to diluted acids because of the direct impact on the handling and transportation costs. However, handling and transportation of concentrated organic acids is extremely hazardous and special security means must be taken to avoid any poisoning risk and injuries. Moreover, the receiving industry must have special storage and adequate managing conditions for these chemicals. In this context, handling and managing of salts of organic acids is suitable and easier because of the high level of safety and facility of storage and handling (Yoo et al. 2010; Luttrell 2012). Although the reactivity of organic acids is higher than that of the conjugated salt, it is possible to convert a salt of an organic acid to the acid form so as to obtain the desired reactivity. In this context, electrochemical activation (or simply “electro-activation”) of salt of an organic acid allows its conversion to the acid form under safe conditions.

Electro-activation is an electrochemical treatment at the electrode/solution interface and is based on the oxidation/reduction phenomena (Aider et al. 2012). The electro-chemical reactions on the electrodes lead to the pH and Redox (oxidation/reduction) potential (ORP) changes as well as formation of highly oxidative species. For example, in the near cathode a surface reduction phenomenon (Eq. 1) involves an abundant liberation of gaseous hydrogen and hydroxyl ions (OH^−^). At the anode surface, oxidation phenomena occurs and high amounts of protonated hydrogen ions (H^+^) are generated following water electrolysis (Eq. 2). These protons can easily react with the salt of an organic acid resulting in the formation of the organic acid form (Sperry and Wright 2006; Palombi et al. 2008; Angamuthu et al. 2010). Thus, from a practical point of view, a food industry that uses organic acids could also use the electro-activation procedure to convert salts of organic acids into their acid form at in-use concentrations for concrete application.

1 $$2 {\text{H}}_{ 2} {\text{O}} + 2 {\text{e}}^{ - } \to \uparrow {\text{H}}_{ 2} + 2 {\text{OH}}^{ - }$$

2 $$2 {\text{H}}_{ 2} {\text{O}} \to \uparrow {\text{O}}_{ 2} + 4 {\text{H}}^{ + } + {\text{ 4e}}^{ - }$$

One of the most effective preservation strategies used in the food industry to ensure food safety and high product quality is the application of antimicrobial treatments by using adequate combination of different and complementary methods (hurdles) (Bari et al. 2005; Rahman et al. 2011). The effect of organic acids obtained after electro-activation of their conjugated salts could provide enhanced antimicrobial activity because of the high reactivity of such solutions which resulted from the excited effect of the applied electric field. Thus, the aim of the present work is to study the electro-activation of three salts of organic acids (potassium acetate, potassium citrate and calcium lactate) in a four compartmental electro-activation reactor modulated by ion exchange membranes in order to generate their conjugated acid form. Impacts of this treatment on the solution’s pH, Redox potential, titratable acidity, Raman spectroscopy and antibacterial properties were studied.

## Methods

### Chemicals and preparations

In this study three salts of organic acids were electro-activated: calcium L-lactate mono-hydrate was purchased from (Sigma-Aldrich, Oakville, Canada). Potassium acetate was purchased from Fisher scientific (Ottawa, Canada) and potassium citrate was purchased from Fisher scientific (Pittsburgh, PA, USA). All used chemicals were of analytical grade. Sodium chloride and sodium hydroxide were purchased from VWR Corp., VWR International (Chicago, IL, USA). Acetic acid (glacial) and citric acid were purchased from Bio Basic Inc., Bio Basic Canada Inc., (Markham, Canada). Lactic acid was purchased from Laboratoire Mat Inc., (Montreal, Canada). Concentrated hydrochloric acid was purchased from Anachemia Co., (Montreal, Canada). All solutions of the studied organic acids salts were prepared the same day the test was performed by dissolving the required amount of powder (crystals) in distilled water to the desired final concentration.

### Design of the electro-activation reactor

**Fig. 1:**
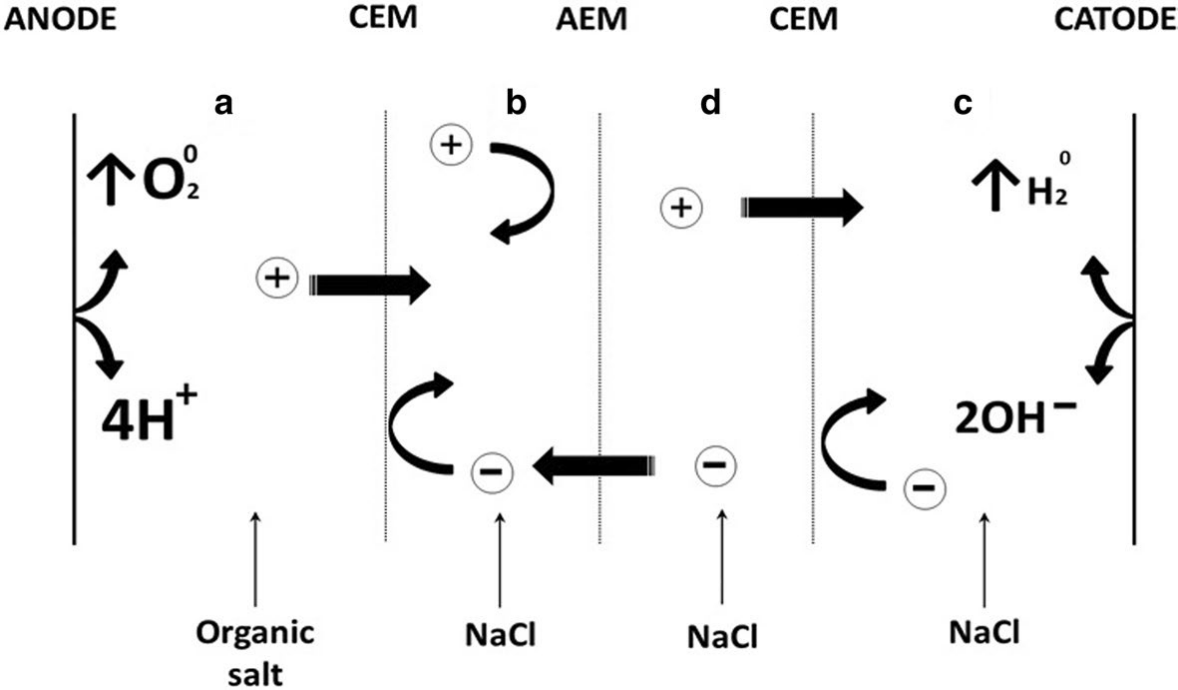
Schematic representation of the used electro-activation reactor. **a** anodic section for anolyte production. **b** section for NaCl acidification by non-contact method. **d** intermediate section. **c** cathodic section for catholyte production. Anions (−) and cations (+) migration across cation exchange (CEM) and anion exchange membrane (AEM)

The general scheme of the electro-activation reactor is presented in Fig. 1. It is composed of four Plexiglas cells divided by two cation (MK-40) and one anion (MA-40) exchange membranes (Shchekinazot, Shchekino, Russia). The diameter of the exchanging areas between the cells is 3 cm, which also corresponds to the diameter of the effective area of the used membranes. The preliminary preparation of membranes was made as described earlier (Liato et al. 2015a). At the opposite ends of a reactor the Ruthenium–Iridium titanium (RuO2–IrO2–TiO2) coated electrodes (Qixin Titanium Co., Ltd, Baoji, Shaanxi, China) were fixed. The anode active area was fixed at 7 cm^2^, while the cathode’s active area was set at 40 cm^2^. The electrodes were connected to direct electric power source (CircuitSpecialists, Tempe, AZ, USA). The potential difference between the electrodes was fixed at 125 V while the amperage was monitored at the generator’s display throughout the electro-activation experiment. Electro-activation was carried out in a batch mode at fixed solutions volume.

### Protocol of electro-activation

Electro-activation treatment was performed in a potentiostatic mode. To avoid the overheating of membranes, the reactor was placed inside a thermostated (refrigerated) bath (Model D-6970, Lauda Brinkmann, Delran, NJ, USA) with forced liquid circulation of 5 L/min. The thermostated bath was filed with water and programmed to keep the temperature at 1 ± 0.5 °C. The temperature in the anodic compartment near the membrane was monitored by electronic thermometer (VWR, Chicago, IL, USA). The anodic chamber was filled with the studied organic acid salt solution of different concentrations (0.1, 0.2, 0.3 % (w/v)) previously cooled to 10 °C. The other compartments of the electro-activation reactor were filled with a 3 % (v/w) NaCl solution (Fig. 1).

The measurements of electro-activated organic solutions (EAOS) main parameters were taken during 180 min of treatment. The pH, total dissolved oxygen (DO) and electric conductivity were monitored by a DO-conductivity-pH meter (SR601C SympHony, VWR, Chicago, IL, USA) with pH-electrode (Orion 8157BNUMD, VWR, Chicago, IL, USA), DO-electrode and conductivity electrode cell (Orion 013005MD, VWR, Chicago, IL, USA), respectively. The measurements of the Redox (Oxidation/Reduction) potential (ORP) was performed with an ORP-meter (Eco Sense ORP15A, YSI Inc., Yellow Springs, OH, USA) calibrated with a ZoBell’s standard solution (Hach Company, Loveland, CO, USA). The titratable acidity was measured by using an automatic titrator (Mettler DL21, Switzerland). A solution of 0.1 M NaOH was fed drop-wise by 0.1 ml until the final point of pH = 7.01 (recognition threshold 20 mV ml^−1^) in the sample solutions was registered by the pH-electrode.

### Raman spectroscopic analysis

All the spectra were recorded on a LABRAM 800HR Raman spectrometer (Horiba Jobin–Yvon, Villeneuve d’Ascq, France) coupled to an Olympus BX30 microscope. The excitation light source of 514.5 nm line was generated by argon ion laser (Coherent, INNOVA 70C Series Ion Laser, Santa Clara, CA, USA). The objectives 10× and 100× MPlan (0.90 NA) were used. For the further samples 100× (0.75 NA) objective (PLF Fluor, Germany) was used. Spectra were recorded from one acquisition of 30 s, the confocal hole and the entrance slit of the monochromator were generally fixed at 200 and 100 µm, respectively. The spectra were corrected using spectral range and a polynomial baseline. Immediately after each electro-activation treatment, an aliquot volume of 15 µl of the studied sample was placed in the microcapillary tubes 1.5–1.8 mm (Chemglass Life Sciences, Vineland, NJ, USA), and then attached to the glass microscope slides. All analyses were repeated three times enabling mean values to be calculated. All experiments were performed at 21 ± 1 °C.

### Bacterial culture and medium

In this study the strain of *E. coli* O157:H7 (ATCC 35150) was used to observe the antibacterial potency of the studied electro-activated solutions. Bacterial culture was obtained from the Food Sciences and Nutrition Culture Collection at Laval University (Quebec, QC, Canada). The used *E. coli* O157:H7 was cultured in Luria–Bertani broth (LB; Difco 244620) at 37 °C for 22 h. Bacterial culture was harvested by centrifugation at 3000×*g* for 15 min at 21 °C, and washed twice with sterile 0.85 % NaCl solution. The final pellet was resuspended in the sterile 0.85 % NaCl solution to a concentration of approximately 10^7^ CFU/ml.

### Sample inoculation and treatments

A 1 mL with 10^7^ CFU/mL *E. coli* cell suspension was mixed with 9 mL of electro-activated solution or sterile salt solution (control) and incubated for 5 min. Immediately after treatment, a 0.1 ml of sample was re-suspended in phosphate buffer solution (0.1 M) to stop the inhibitory effect of the electro-activated solutions. To determine the number of viable survivors, a 10-fold serial dilution with sterile salt solution was performed, whereby samples were spread-plated onto LB agar and incubated at 37 °C for 24 h to enable viable counting. Diluted samples were spread-plated onto LB agar and incubated at 37 °C for 24 h before counting. The combination of treatments were performed by dilution the electro-activated anolytes with acidified NaCl solutions to get a final concentration of 0.1–0.2 %. To do this, appropriate volumes of electro-activated solutions of potassium acetate, potassium citrate and calcium lactate were mixed with corresponding volumes of acidified NaCl solution of the adjacent compartment to the anolyte section. The salt of NaCl was diluted into the sample solution to reach the final concentrations and the pH of all tested solutions was adjusted to the value 2.6 ± 0.36 by the addition of HCl/NaOH (0.5 M). The antimicrobial efficacy of these combination treatments was assessed as described above.

### Statistical analysis

This work was performed by using a full factorial experimental design. All experiments were carried out in triplicates and the mean values ± standard deviation (SD) were recorded and used for comparisons. The obtained data were analyzed to investigate the differences between the mean values at 95 % confidence level by using a One-way analysis of variance (ANOVA) procedure, and plotted by using the Systat-10 Software (Systat Software, Inc., San Jose, CA, USA).

## Results

### Evolution of pH in the electro-activated solutions

**Fig. 2:**
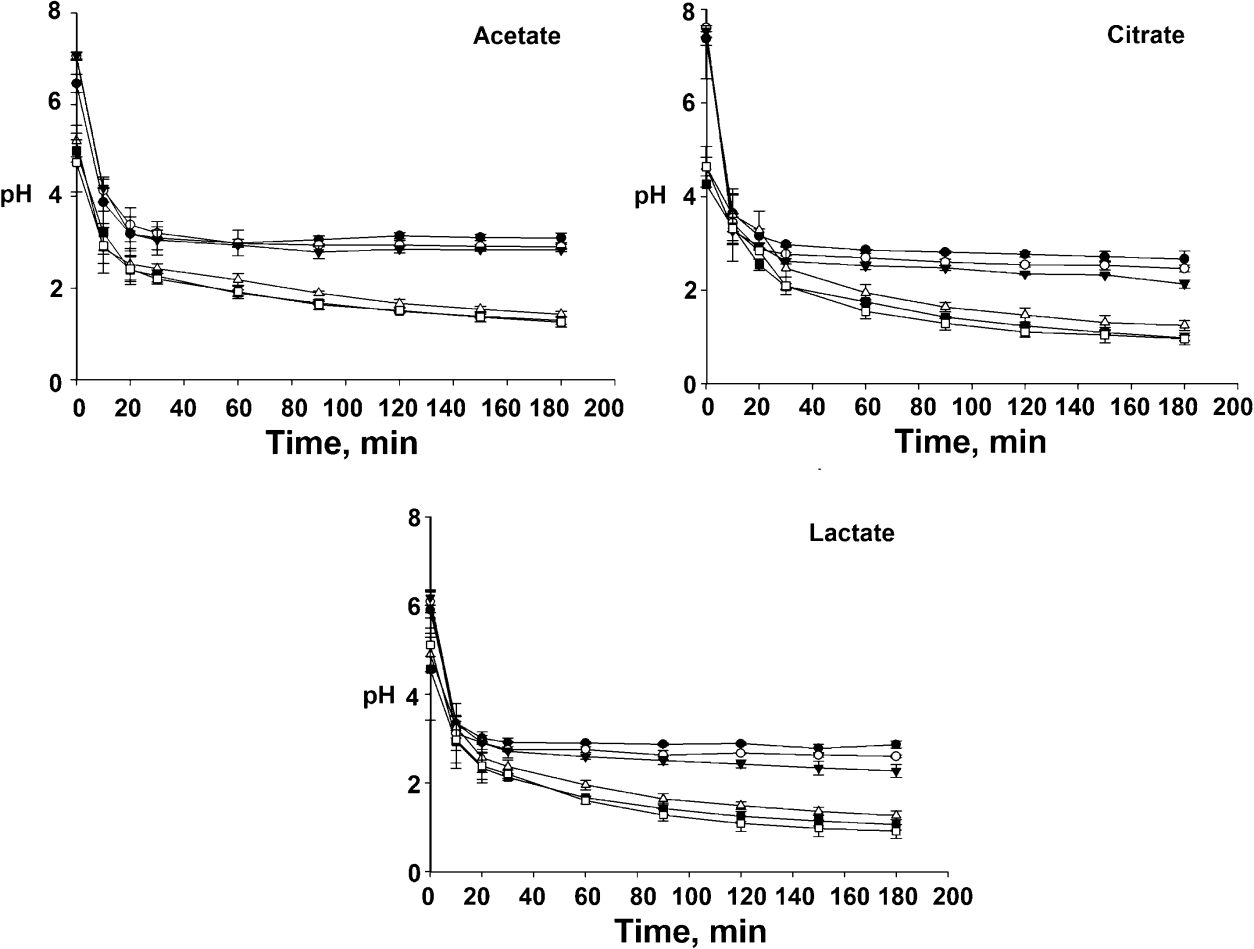
pH variation of the anolytes of the electro-activated salts of the organic acids (*filled circle* 0.1,* open circle* 0.2,* inverted filled triangle* 0.3 %) and acidified NaCl solutions (3 %) with corresponding anolyte concentrations (*open triangle* 0.1, *filled square* 0.2, *open square* 0.3 %)

The most important changes of pH in electro-activated organic solutions (EAOS) are due to water electrolysis reactions on the electrodes (Eqs. 1, 2). Figure 2 showed the drastic changes from slightly acidic or neutral pH to highly acidic pH in the solutions of the anodic (anolyte) and the adjacent chamber to the anodic compartment (acidified NaCl solution). All organic salt solutions (OSS) as well as the acidified NaCl solution, had pH below 4, after 10 min of treatment. There was no statistical difference between the different concentrations of OSS. However, the solutions at 0.3 % concentration showed the lowest pH. The type of OSS was found to be significant factor affecting the acidity and OSS could be ordered as follows: citrate < lactate < acetate from the most acid to the least acid. At the end of the electro-activation treatments, the obtained solutions with initial concentration of 0.1 % reached the following pH values: 2.66 ± 0.18, 2.86 ± 0.07 and 3.09 ± 0.01, respectively.

### Titratable acidity and buffering capacity of the electro-activated solutions

**Fig. 3:**
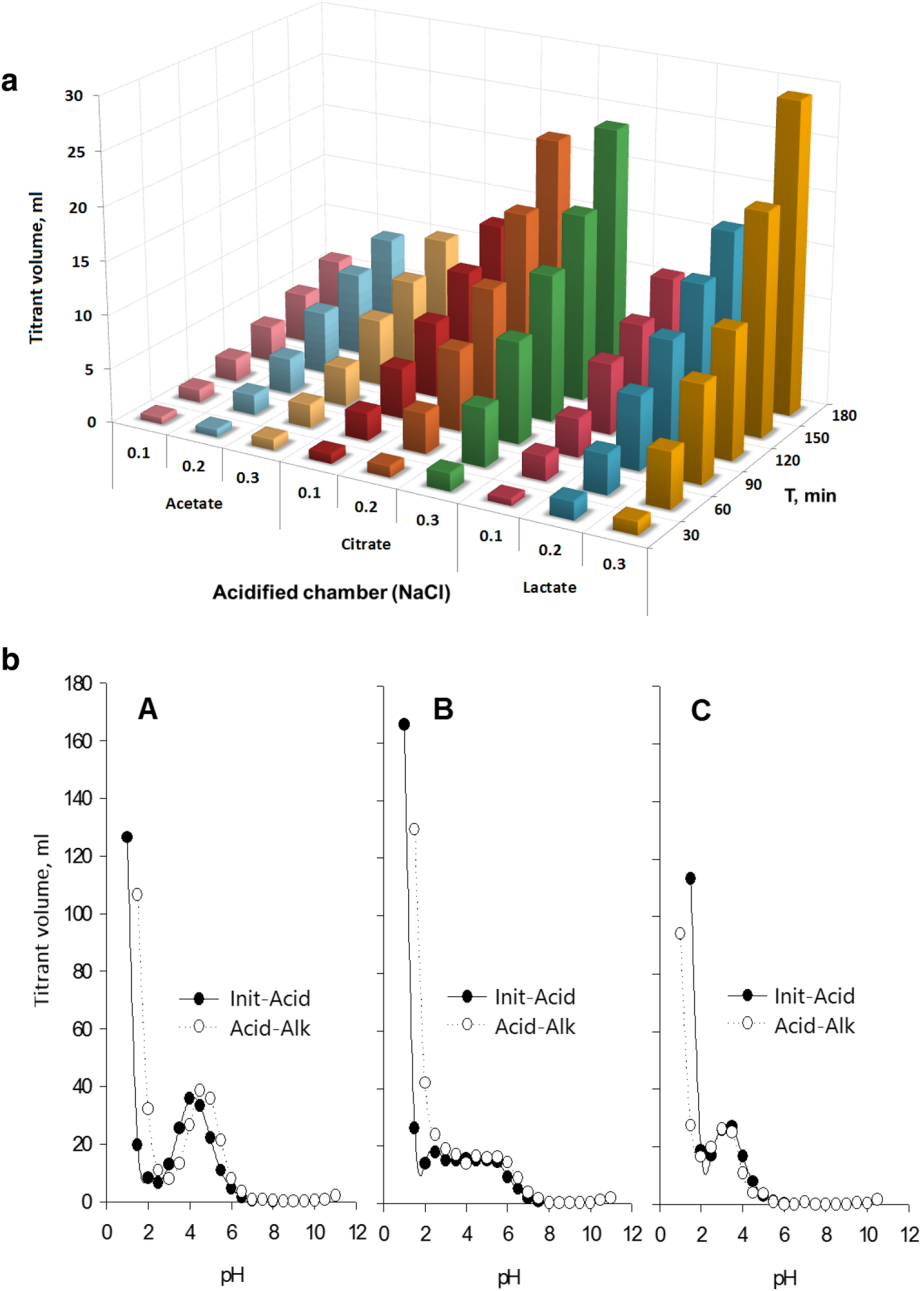
**a** Variation of the titratable acidity of NaCl solutions in the acidifying section by a non-contact mode. **b** Buffering capacity of the electro-activated solutions of: potassium acetate (*A*), potassium citrate (*B*) and calcium lactate (*C*)

The results of the titratable acidity (TA) of the anolytes (Fig. 3a) and the solutions buffering capacity (Fig. 3b) summarize the effect of the electro-activation treatment on solutions acidity. During the electro-activation treatment, the TA of the anolytes were observed to be unchangeable after 30 min of treatment. At the same time, the pH of the electro-activated solutions of the salts of organic acids significantly decreased (Fig. 2). This phenomenon was caused by the properties of the used salts of weak organic acids which are known as weakly dissociating chemicals. In this case, only a few amount of ionizable hydrogen ions are dissociated and continue to maintain the buffering capacity. The obtained results showed that the buffering capacity of the electro-activated solutions at a 0.3 % concentration was dependent of the type of the electro-activated salt of organic acid (Fig. 3b). The electro-activated solution of potassium acetate had the highest buffering capacity at the pH range between 4 and 3, where its pH was stable during the first 20 min of the electro-activation treatment. It was followed by the buffering capacity of the electro-activated calcium lactate and potassium citrate which have no significant difference between them (p < 0.001). Finally, it is necessary to mention that the electro-activated solution of the used salts of organic acids have a strong buffering capacity when the pH was below 2.5.

### Effect of electro-activation on Redox potential, current intensity and temperature variation

**Fig. 4:**
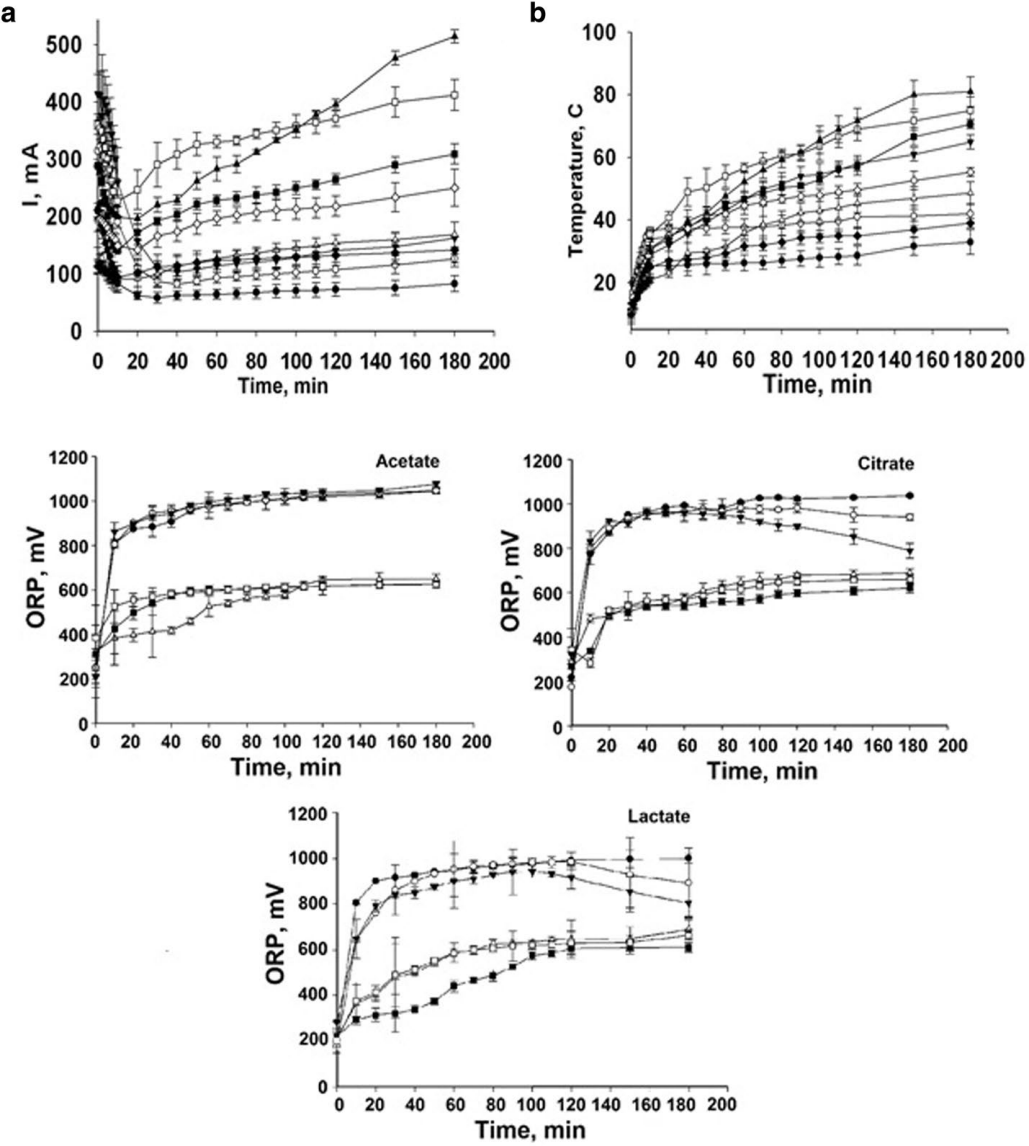
The variation of current intensity (**a**), temperatures (**b**) and ORP of the anolytes under different concentrations of anolyte (*filled circle* acetate 0.1 %, *open circle* acetate 0.2 %, *inverted filled triangle* acetate 0.3 %, *open triangle* citrate 0.1 %, *filled square* citrate 0.2 %, *open square* citrate 0.3 %, *filled diamond* lactate 0.1 %, *open diamond* lactate 0.2 %, *filled triangle* lactate 0.3 %) during electro-activation

The changes in the oxidation–reduction potential (ORP) showed that the anolytes of OSS were found in highly oxidative state (Fig. 4). The results showed that at the end of the EA treatment the ORP of 0.1 % (w/v) OSS of potassium acetate, potassium citrate and calcium lactate increased from +250 ± 23 to +1046 ± 14.46, +1035 ± 5.13 and +999 ± 21.3 mV, respectively. The most important changes in the properties of electrolyzed solutions, including ORP, occur near solution-electrode interfaces in a thin electric double layer (Aider et al. 2012).

Regarding the evolution of electric current intensity during the solutions electro-activations, the obtained data showed that during the first 5 min of electro-activation, the current dropped down. This behavior indicated occurrence of some electric resistance at the beginning of the electro-activation process. However, after 10 min of electro-activation, in all cases, we observed a significant increase of the electric current intensity, indicating that the electro-activation system is highly conductive and the occurred electric resistance at the beginning of the process was completely disappeared. This behavior is technologically favorable since it is a contributing factor for low power consumption.

The temperature varied as a function of the used treatment. In all cases, we observed an increase of the temperature which ranged from 30 to 80 °C. It was mainly caused by the Joule heating at the electrode/solution interface. Generally, the highest the electric conductivity of the system, the lower the temperature increase is. Moreover, from technological point of view, the Joule heating can be exploited as source of solution heating to improve their antibacterial effect.

### Raman scattering light spectra of anolytes

**Fig. 5:**
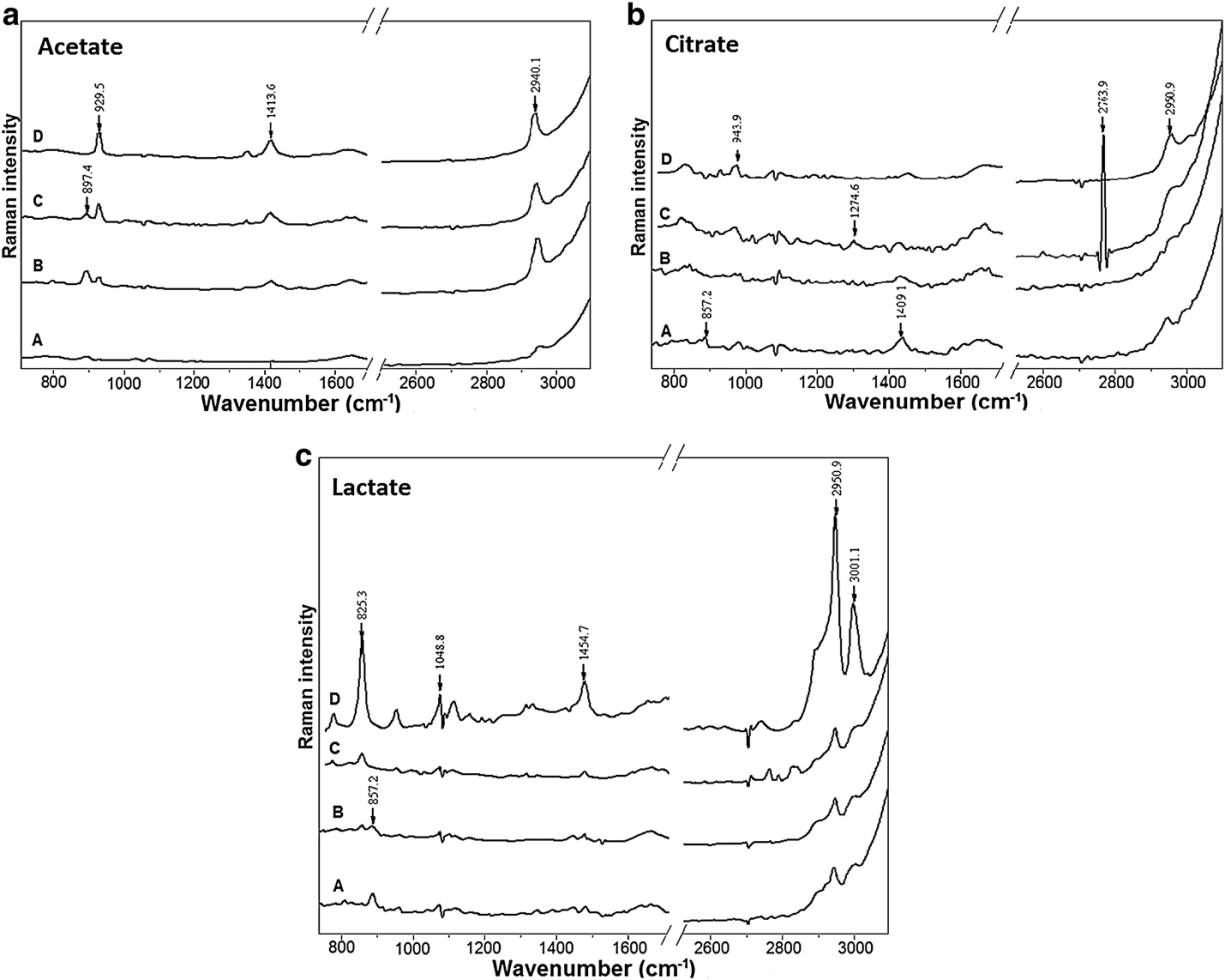
Raman spectra of the 0.3 % electro-activated solutions of the used salts of weak organic acids: (*A*) non-treated solution, (*B*) after 15 min of electro-activation, (*C*) after 30 min of electro-activation, (*D*) a control corresponding to the commercial conjugated acid of the used salt of the organic acid. **a** Potassium acetate solution, **b** potassium citrate solution, **c** calcium lactate solution

The results of Raman spectra of the non-treated and EA treated anolytes of OSS are shown in the Fig. 5. The samples with 0.3 % concentration were taken after 15 and 30 min of treatment when their properties had the optimal value of pH and ORP. The scattering intensity of anolytes was found in the 2950.9 cm^−1^ peak which is also corresponding to the peak of organic acids. The water electrolysis contributes to H^+^ accumulation which is leads to acid properties of these solutions and conversion of salts from their conjugated forms. However spectra of acidified NaCl solutions didn’t show any difference compared with the control sample (H_2_O) (data not shown).

### Antimicrobial activity of electro-activated solutions

The results on the effect of the electro-activated solutions on *E. coli* O157:H7 are summarized in Table 1. The obtained data on population survival of *E. coli* O157:H7 demonstrated a high antibacterial effect of the electro-activated solutions of the used salts of weak organic acids; particularly potassium acetate and calcium lactate. A lower antimicrobial effect in comparison with these two solutions was demonstrated by the electro-activated potassium citrate solution. For this treatment, the anolytes of the electro-activated solutions of salts of organic acids were generated during 30 min and acidified NaCl solutions in the adjacent compartment to the anolyte section were obtained after 180 min of EA, using lactate salt as anolyte, as aforementioned. Results of the inactivation treatment of the inoculum by anolytes of the potassium acetate showed complete reduction of the *E. coli* O157:H7 population which had an initial concentration of 6 ± 0.06 log of CFU/ml. Moreover, the obtained data showed that although the anolyte obtained after electro-activation of calcium lactate alone had less pronounced bactericidal impact than potassium acetate, when it was combined with 1.5 % of acidified NaCl solution, it exhibited a reduction effectiveness of ≥6 log CFU/ml of *E. coli* O157:H7 population. The same tendency was observed for the anolyte obtained after electro-activation of potassium citrate, where its combination with 1.5 and 3 % acidified NaCl solution significantly reduced the bacteria surviving concentration of *E. coli* O157:H7 (Table 1). Moreover, it is worthy to mention that non-treated NaCl solutions as well as non-activated used salts of the three organic acids have no antimicrobial effect on the bacteria while the acidified NaCl solutions by non-contact electro-activation mode showed a 3.75 ± 0.22 and 1.84 ± 0.24 log CFU/ml surviving of *E. coli* O157:H7 for 1.5 and 3 % solutions concentrations, respectively. This observation is an evident demonstration of the antimicrobial effect of both electro-activated organic salts and the electro-activated NaCl solution by the non-contact mode. Furthermore, the combination treatment of the anolytes of OSS with acidified NaCl showed a high reduction of *E. coli* O157:H7, which demonstrate strong synergistic effect (Table 1).

**Table 1:** Surviving concentration of *E. coli* O157:H7 in electro-activated solutions of salts of weak organic acids alone or in the combination with acidified NaCl solution during 5 min of treatment

Treatment		Reduction (log CFU/ml)		
		100 % anolyte	Anolyte + 1.5 % acidified NaCl	Anolyte + 3.0 % acidified NaCl
Potassium acetate, %	0.10	ND	ND	ND
	0.15	ND	ND	ND
	0.20	ND	ND	ND
Potassium citrate, %	0.10	5.83 ± 0.11	3.70 ± 0.18	1.84 ± 0.24
	0.15	5.71 ± 0.23	3.75 ± 0.22	ND
	0.20	5.43 ± 0.32	3.46 ± 0.16	ND
Calcium lactate, %	0.10	4.17 ± 0.16	ND	ND
	0.15	4.02 ± 0.18	ND	ND
	0.20	2.57 ± 0.23	ND	ND

*ND* negative by enrichment and no detectable survivors by a direct plating procedure

Initial *E. coli* O157:H7 population was 6 log CFU/ml

## Discussion

*Evolution of pH* During the electro-activation of OSS, the reactions of a decarboxylative dimerisation (Eq. 3) of two carboxylate ions, known as Kolbe reactions, could additionally occur in the system. The Kolbe electrolysis is the main reaction at the anode surface and is used for the oxidation of organic salts, producing methyl radicals with CO_2_, and subsequent formation of dimethyl radical (Smith and Gilde 1961):

3 $$2 {\text{RCOO}}^{ - } \to {\text{ 2CO}}_{ 2} + {\text{ R}} - {\text{R }} + {\text{ 2e}}^{ - }$$

The application of small OSS concentrations impair the release of dimethyl, hence it may promote water electrolysis (Svadkovskaya and Voitkevich 1960). Used concentration of OSS demonstrates significant changes of the anolyte pH. In addition, the high concentration of dissolved oxygen (DO) (25 ± 3.53 mg/L) was found for all anolytes after 30 min of electro-activation treatement demonstrating active electrolysis on the anode/solution interface. The majority of the related works are devoted on the mechanisms of the CH3• radical production, while others are focused to the application of NaCl solutions as anolyte (Smith and Gilde 1961). However, few works on the electro-activation of salts of organic acids reported significant pH decrease to values of 4.0/5.5 with concomitant increase in total dissolved oxygen (DO) up to 7–15 mg/L (Osadchenko et al. 2008, 2009). The NaCl solution of the acidified chamber (adjacent to the anodic compartment) was also characterized by significant decrease of the pH. Due to active anode electrolysis and proton migration through the cation exchange membrane MK-40 (Fig. 1), the pH of this solution fell below 4 at the same time as anolyte of OSS. However, in contrast to anolyte the acidified NaCl solution reached the pH value less than 1, at the end of treatment. It was found that the acidity of NaCl solution followed the same order as was previously described for OSS type (citrate < lactate < acetate) and OSS concentrations (0.3 < 0.2 < 0.1 %). For example, at the end of electro-activation treatment, the pH of salt solutions of potassium acetate as anolyte with concentrations from 0.3 to 0.1 % were 1.25 ± 0.10, 1.29 ± 0.08 and 1.42 ± 0.06, respectively. These results were found in accordance with previous studies in three-compartment reactor, however NaCl concentration influenced the pH of acidified solution and varied from ca. 2 to 10 after 1 h of treatment (Liato et al. 2015a) (Fig. 2). In this study, the configuration of the reactor enabled creation of two types of acidified solutions. The anolyte was obtained in the anodic compartment as a result of the oxidation–reduction phenomena at the anode/solution interface (so-called anolyte in this study). The acidified NaCl solution by contactless electro-activation is the solution which was obtained in the adjacent compartment to the anodic side, as shown in Fig. 1. On one hand the anolyte of OSS decreased its pH to 3 and less, on the other hand acidified NaCl solutions decreased to pH less than 1. For example, the salts of acetate, citrate and lactate with concentration of 0.3 % after 180 min of EA treatment reach the pH 2.13 ± 0.08, 2.26 ± 0.14 and 2.82 ± 0.03 at the end of treatment, respectively. The acidified NaCl solutions of OSS under the same conditions decrease pH to 1.25 ± 0.10, 0.95 ± 0.12 and 0.90 ± 0.15, respectively. Obtained results show that the acidified NaCl solutions form strong acid (like HCl) which may fully disassociate and give low pH values (e.g. pH 1 and less). In contrast, electro-activated OSS do not dissociate completely, thus their pH would not be as low (Sadler and Murphy 2010). As it is known the pH demonstrates only the equilibrium measure of the hydronium ion (H_3_O^+^) concentration in aqueous solutions, whereas the titratable acidity (total acidity) denotes the overall acid concentration (Fig. 3a).

*Titratable acidity* According to the Henderson–Hasselbalch equation (Eq. 4), the value of pH is a sum of acid dissociation constant ($pK_{a}$) and the relation of conjugated base to its concentration of undissociated acid ($[A^{ - } ]/\left[ {HA} \right]$). The thermodynamic value of the $pK_{a}$ characterizes the equilibrium (Eq. 5) of the proton transfer from the acid–base equilibrium and related to the concentration and type of the organic salt (Sadler and Murphy 2010). The shift in acid–base equilibrium of the OSS anolytes was, thereby, protonated during electro-activation and remained in the stable acid form. In addition, the acid–base equilibrium of anolytes was also maintained by the proton migration and acidfication of adjacent compartment (Fig. 3a, b).

4 $${\text{pH}} = {\text{p}}K_{a} + { \log }\frac{{[A^{ - } ]}}{{\left[ {HA} \right]}}$$

5 $${\text{HA}} \leftrightarrow {\text{ H}}^{ + } + {\text{A}}^{ - }$$

The TA of acidified NaCl solutions were found to be significantly higher than TA of anolyte solutions (Fig. 3a). The main cause is the proton migration toward the cathode through the cation exchange membrane and the buffer capacity of OSS, as discribed previously. At the end of treatment when 0.3 % solution of potassium acetate, potassium citrate and calcium lactate were used in the anodic compartment, the NaCl solutions in the acidified chamber were titrated to the pH 7 with 10.20 ± 1.89, 23.73 ± 9.97 and 29.14 ± 5.91 ml of titrant, respectively (Fig. 2). The time was the most significant factor for TA evolution of the acidified NaCl solutions due to the anode electrolysis. However, the type of anolyte was also found significant. It was observed that TA evolution of acidified NaCl solutions depended on the current intensity and temperature changes of the anolytes during the EA treatment (Fig. 4). The results show that the OSS of lactate and citrate have the most significant changes during the EA treatment, which is in correspondence with the results of TA. The OSS used in the present study differ by their molecular characteristics which may explain the obtained results. Comparing the molar mass of OSS, one may conclude that higher concentration of potassium acetate promotes better electron transfer and consequently this phenomenon results in less water electrolysis. Regarding the conditions appeared in the reactor (Fig. 1), Kolbe reaction competes with water electrolysis leading to the lesser Joule heating (Engelhardt and Eger 1934; Bagotsky 2005). Thus, the temperature for potassium acetate remains below other OSS anolytes and therefore, the TA of acidified NaCl solution showed significantly lower value than other OSS (Fig. 3a). When potassium citrate was used to generate the desired anolyte, the TA of the acidified NaCl solution in the adjacent section was not statistically different from the TA of the acidified NaCl solution used in the treatment for the electro-activation of a calcium lactate solution. However, as a calcium lactate solution, the potassium citrate solution exhibited important increase in temperature and current intensity during the electro-activation treatment (Fig. 4). This factor is very important since temperature promotes hydrogen bond rupture and hence better electrolysis for both types of salts (Bagotsky 2005). It is worth noting, that the molecule of potassium citrate has no radical branches like potassium acetate or calcium lactate salts, thus Kolbe reaction does not take place and the water electrolysis is more efficient. Moreover, calcium lactate dissociates weakly compared to potassium acetate, thus the electrolysis is better. This process consequently promotes proton migration and further acidification of NaCl solution in the adjacent chamber. It is also important to mention that the mass transfer of ions of the OSS in stationary conditions depends on the molecular mass, thus the least mobile OSS are citrate potassium (the highest molecular mass) followed by calcium lactate and potassium acetate.

*Redox potential* The reactions on the electrodes are mainly due to water electrolysis including a formation of small amount of highly reactive radicals (H^•^, H_2_, OH^•^, ^1^O_2_, O_3_, H_2_O_2_, O_2_^•−^) (Prilutsky 1999; Gnatko et al. 2011; Chaplin 2006). The reactions of water electrolysis and radical formation at the electrode surface (in the presence of a direct electric current) (Eq. 6–12) give the energy to the solutions and create its oxidative/reduced state (Chaplin 2006). Electrode interaction with the OSS may also have radical combination with some of the metastable species generating CH_3_OO^•^ radicals (Fernández‐Castro et al. 2015).

6 $$2 {\text{H}}_{ 2} {\text{O}} \to {\text{O}}_{ 2} + 4 {\text{H}}^{ + } + {\text{ 4e}}^{ - } \left( {{\text{E}}^{\text{o}}_{\text{ox}} = + 1. 2 3 {\text{V}}} \right)$$

7 $$2 {\text{H}}_{ 2} {\text{O}} \to {\text{O}}_{ 3} + 2 {\text{H}}^{ + } + {\text{ H}}_{ 2} {\text{O }} + {\text{ 4e}}^{ - } \left( {{\text{E}}^{\text{o}}_{\text{ox}} = + 2.0 8 {\text{V}}} \right)$$

8 $$2 {\text{H}}_{ 2} {\text{O}} \to {\text{H}}_{ 2} {\text{O}}_{ 2} + 2 {\text{H}}^{ + } + {\text{ 2e}}^{ - } \left( {{\text{E}}^{\text{o}}_{\text{ox}} = + 1. 7 8 {\text{V}}} \right)$$

9 $${\text{H}}_{ 2} {\text{O}}_{ 2} \to {\text{O}}_{ 2} + 2 {\text{H}}^{ + } + {\text{ 2e}}^{ - } \left( {{\text{E}}^{\text{o}}_{\text{ox}} = + 0. 6 8 {\text{V}}} \right)$$

10 $${\text{H}}_{ 2} {\text{O}}_{ 2} \to {\text{ HO}}_{ 2}^{ \bullet } + {\text{H}}^{ + } + {\text{ e}}^{ - } \left( {{\text{E}}^{\text{o}}_{\text{ox}} = + 1. 50 {\text{V}}} \right)$$

11 $${\text{H}}_{ 2} {\text{O}} \to {\text{HO}}_{ 2}^{ \bullet } + {\text{H}}^{ + } + {\text{ e}}^{ - } \left( {{\text{E}}^{\text{o}}_{\text{ox}} = + 2. 4 3 {\text{V}}} \right)$$

12 $${\text{H}}_{ 2} {\text{O}} \to \bullet {\text{O}} \bullet \, + 2 {\text{H}}^{ + } + {\text{ 2e}}^{ - } \left( {{\text{E}}^{\text{o}}_{\text{ox}} = + 2. 4 3 {\text{V}}} \right)$$

In our study it was found that ORP values of the EA treatment of potassium acetate (0.3 %) increased significantly reaching 1076 ± 12 mV in the end of the EA treatments, which was in agreement with other studies (Osadchenko et al. 2008, 2009; El Jaam et al. 2016). The ORP values of the potassium citrate and calcium lactate slightly decreased after 60 and 90 min of EA treatment, respectively. For example, ORP of electro-activated potassium citrate (0.2 and 0.3 %) after 1 h of treatment decreased from +966 ± 20 and +959 ± 38 mV down to +939 ± 14 and +788 ± 33 mV, respectively. In contrast to our data El Jaam (2016) reported that the ORP of EA citrate potassium salt was not significantly increased during 1 h of treatment (418 ± 15 mV) (El Jaam et al. 2016) (Fig. 4). The maximum ORP value of OSS of calcium lactate (0.2 and 0.3 %) was reached after 90 min of electro-activation with mean values of +984 ± 11 and +941 ± 48 mV, respectively. But it decreased to +852 ± 15 and +803 ± 28 mV by the end of the treatment. This phenomenon is due to the properties of the potassium citrate and calcium lactate related to Kolbe reactions, in contrast to potassium acetate. Electric treatment that was performed at a potentiostatic mode substantially affected the electric current intensity, as aforementioned. The increase of the temperature influenced the hydrogen bonds of the H_2_O and subsequently enhanced the water splitting, resulting in more electric current carriers which were deficient in the OSS solutions (Engelhardt and Eger 1934). Thus, according to the Nernst equation in real conditions (not ideal) (Eq. 13), a temperature increase due to the passage of electric current (Joule effect) leads to ORP decrease (Bagotsky 2005) (Fig. 4).

13 $$E = E_{0} - \frac{RT}{NF}{ \log }\frac{{\left[ {Red} \right]}}{{\left[ {Ox} \right]}}$$

The results of acidified NaCl solutions showed the increase of the ORP during the EA treatment, showing that these salt solutions are transformed into a highly oxidative state (Fig. 4). The ORP of acidified NaCl solutions changed from ca. +320 ± 37 to +650 ± 23 mV at all OSS and at all concentrations. The impact of the type and concentration of OSS was not significant for the solutions in the acidified chamber. However, the results of our previous study showed that salt concentration and current intensity were the most significant factors influencing the ORP of solutions in the acidified NaCl solution (Liato et al. 2015a). In contrast with the anolytes, the acidified NaCl solutions, which were electro-activated without direct contact with the electrode (anode), the ORP changed through a different pathway. The ORP changes in aqueous electrolytes are a complex process involving also the reactions of H^+^ and OH^−^ ions. According to the Nernst equation, ORP depends on the concentration of these ions (or more accurately, ion’s activity) determined by the potential difference between two electrodes (Eq. 14). Hence, ORP is related to the pH value of monovalent ions. When pH is raised by 1 unit, the Redox potential becomes hereby 0.059 V more negative, and vice versa (Bagotsky 2005). Thereby the ORP of acidified NaCl solutions increases while the pH decreases (Fig. 2).

14 $$E = E_{0} + 2.303\frac{RT}{NF}{ \log }A$$

Where *E*—measured electrode potential, $E_{0}$—standard electrode potential, *R*—universal gas constant, *F*—Faraday constant, *T*—absolute temperature, *N*—ion’s number of charges, and *A*—activity of the ion.

*Raman scattering light spectra* A difference in the peak intensity of the anolyte was noted in the region 3410–3430 cm^−1^. This region generally corresponds to stretching vibrations of the hydrogen-bonded OH, so called Fermi resonance (Busing and Hornig 1961; Murphy and Bernstein 1972). The changes in the intensity in this region has also been observed in other works suggesting that this is also may due to the changes in electrolyte concentration or different enthalpies of the solutions caused by temperature (Pernoll et al. 1975; Maréchal 2011). The Raman spectra intensity of the electro-activated solutions were significantly different from those of the commercial solutions of organic acids and the non-treated solutions of the salts of the used organic acids (Fig. 5). For example the anolyte of acetate potassium showed scattering intensity at the regions of 874 cm^−1^, which is not significantly different from the non-treated acetate and aligned at the control solution (acetic acid). The different results in Raman shifts appeared at the treated anolytes of OSS could be the result of solution’s composition caused by reactions at the electrode surface or OSS conversions to other species. More research needed to explain the phenomena of electro-activation treatment. Some publications on the investigations of Raman scattering of the anolyte of NaCl solutions showed that EA treatment has important impact on water properties (Pastukhov and Morozov 2000). It was reported that the intensity changes in the spectral region between 700 and 2700 cm^−1^ showed the presence of a charged hydrogen bonds which could be associated to the excessive presence of H^+^ and OH^−^ ions (Pastukhov and Morozov 2000). In contrast to reported study our configuration consisted of a four-cell electro-activation reactor (Fig. 1) that generally allows the passage of the protons which also could be the principal factor of water clustering (Xiong et al. 2010; Aider et al. 2012). Moreover, the concentration of OSS is one of the significant factors for water electrolysis, higher concentrations cause more pronounced changes in the properties of EA solutions that may provide more pronounced scattering intensity (Leonov et al. 1999; Gerzhova et al. 2015).

*Antimicrobial activity E. coli* O157:H7 as a member of the enterohemorrhagic group of pathogenic bacteria frequently occurs as a foodborne and waterborne pathogen and is a major public health concern (Lee and Kang 2009). The present work devoted to the inactivation of the O157:H7 strain of *Escherichia coli* showed that the treatment with electro-activated aqueous solutions of food grade organic salts is highly efficient under the conditions of the assay performed (Table 1). Moreover, the results showed that electro-activation of such solutions creates specific synergistic conditions with a stronger effect than conventional hurdle approaches used to ensure microbial food safety (Huang et al. 2008; Aider et al. 2012). Thus, it may be suggested that appropriate application of this treatment allows the reduction of 6 log CFU/ml of bacteria within 5 min contact time, especially when a combination treatment is used (Huang et al. 2008). Application of the organic acids in combination with different treatments like heat or salt has been commonly used to ensure the microbial safety of foods in many products. The combined treatment can result in additive results, such as additive, synergistic, and antagonistic effects (Lee and Kang 2009). However, although the organic acids are effective against different pathogens, they are usually supplied in a concentrated form. Thus, they must be handled with care to ensure adequate safety of the employees. Moreover, acids in strong concentration from should be used with high precaution because they may have undesirable effects on the sensory quality of the food product. Thus, it seems to be preferable to use low concentration of organic acid solutions during the combination treatment (Choi et al. 2009; Luttrell 2012). The anolytes of OSS during the EA treatment acquire important changes in properties which are able to disturb the bacteria homeostasis and act synergistically with heat or other hurdles (Liato et al. 2015b). On one hand, the growth of micro-organisms is mostly dependent on the medium acidity; namely the solution pH and its titratable acidity which is a measure of all free and bond hydrogen ions available to maintain a buffering capacity of acidic solution. Moreover, the balance of oxidizing and reducing agents may significantly affect the environmental conditions in which the bacteria are growing. This parameter called the oxidation–reduction potential (ORP) of the solution is intensively studied and was used as effective parameter for microbial control and it is estimated as one of the crucial parameters for satisfactory biotic homeostasis (Lund and Wyatt 1984; Bagramyan et al. 2000). The creation of the adverse conditions through extreme values of the ORP requires the application of additional chemical agents which could be eliminated by using an electro-activation reactor (Aider et al. 2012). Moreover, the oxidative radicals generated by electro-activation is also considered as a source of the antimicrobial effect of electro-activated solutions. This particularity was successfully exploited for use forn food safety and in medicinal practice as preservative or desinfectant (Aider et al. 2012; Al-Haq and Gómez-López 2012; Gil et al. 2015). Related works on electro-activated solutions showed that the NaCl is the most utilised salt due to the generation of strong chlorine radicals (Liato et al. 2015a). Its radical combinations resulting from electro-activation treatment were found to be more significant for microbial reduction in comparison with hydroxyl radicals (Hao et al. 2012). However hydroxyl radicals are the most reactive radicals and could be the principal starters of chlorine reactivity. Although this phenomenon creates adverse conditions for microorganisms growth, it could also be harmful for human. Thus, it is considered as undesirable agent for food preservation (Rico et al. 2007; Xiong et al. 2010). The oxygen species, pH and especially ORP are additional factors which influence the antimicrobial activity of EA solutions and explain the effectiveness of the electro-activation technology as strong desinfection tool (Kim et al. 2000; Liao et al. 2007). Thus, organic acids obtained by electro-activation of their conjugated salts, as inexpensive and environmentally friendly compounds, can be successfully used for food preservation.

## Conclusions

In this work, we demonstrated that electro-activation is highly effective to convert aqueous solutions of salts of weak organic acids (potassium acetate, potassium citrate and calcium lactate) to highly reactive solutions with strong antibacterial effect against *E. coli* O157:H7. Moreover, Raman spectra showed that these solutions were quite different from the commercial solutions of acetic, citric and lactic acids. This could be attributed to the high excited level of the electro-activated components. Finally, this study opens possibilities to develop safe, nutritive and low heat processed foods.
